# Decision Tree Algorithms Predict the Diagnosis and Outcome of Dengue Fever in the Early Phase of Illness

**DOI:** 10.1371/journal.pntd.0000196

**Published:** 2008-03-12

**Authors:** Lukas Tanner, Mark Schreiber, Jenny G. H. Low, Adrian Ong, Thomas Tolfvenstam, Yee Ling Lai, Lee Ching Ng, Yee Sin Leo, Le Thi Puong, Subhash G. Vasudevan, Cameron P. Simmons, Martin L. Hibberd, Eng Eong Ooi

**Affiliations:** 1 Novartis Institute for Tropical Diseases, Singapore; 2 Tan Tock Seng Hospital, Singapore; 3 Genome Institute of Singapore, Singapore; 4 National Environment Agency, Singapore; 5 Dong Thap Hospital, Cao Lanh, Dong Thap Province, Vietnam; 6 Oxford University Clinical Research Unit, Hospital for Tropical Diseases, Ho Chi Minh City, Vietnam; 7 DSO National Laboratories, Singapore; Oxford University Clinical Research Unit, Viet Nam

## Abstract

**Background:**

Dengue is re-emerging throughout the tropical world, causing frequent recurrent epidemics. The initial clinical manifestation of dengue often is confused with other febrile states confounding both clinical management and disease surveillance. Evidence-based triage strategies that identify individuals likely to be in the early stages of dengue illness can direct patient stratification for clinical investigations, management, and virological surveillance. Here we report the identification of algorithms that differentiate dengue from other febrile illnesses in the primary care setting and predict severe disease in adults.

**Methods and Findings:**

A total of 1,200 patients presenting in the first 72 hours of acute febrile illness were recruited and followed up for up to a 4-week period prospectively; 1,012 of these were recruited from Singapore and 188 from Vietnam. Of these, 364 were dengue RT-PCR positive; 173 had dengue fever, 171 had dengue hemorrhagic fever, and 20 had dengue shock syndrome as final diagnosis. Using a C4.5 decision tree classifier for analysis of all clinical, haematological, and virological data, we obtained a diagnostic algorithm that differentiates dengue from non-dengue febrile illness with an accuracy of 84.7%. The algorithm can be used differently in different disease prevalence to yield clinically useful positive and negative predictive values. Furthermore, an algorithm using platelet count, crossover threshold value of a real-time RT-PCR for dengue viral RNA, and presence of pre-existing anti-dengue IgG antibodies in sequential order identified cases with sensitivity and specificity of 78.2% and 80.2%, respectively, that eventually developed thrombocytopenia of 50,000 platelet/mm^3^ or less, a level previously shown to be associated with haemorrhage and shock in adults with dengue fever.

**Conclusion:**

This study shows a proof-of-concept that decision algorithms using simple clinical and haematological parameters can predict diagnosis and prognosis of dengue disease, a finding that could prove useful in disease management and surveillance.

## Author Summary

Dengue illness appears similar to other febrile illness, particularly in the early stages of disease. Consequently, diagnosis is often delayed or confused with other illnesses, reducing the effectiveness of using clinical diagnosis for patient care and disease surveillance. To address this shortcoming, we have studied 1,200 patients who presented within 72 hours from onset of fever; 30.3% of these had dengue infection, while the remaining 69.7% had other causes of fever. Using body temperature and the results of simple laboratory tests on blood samples of these patients, we have constructed a decision algorithm that is able to distinguish patients with dengue illness from those with other causes of fever with an accuracy of 84.7%. Another decision algorithm is able to predict which of the dengue patients would go on to develop severe disease, as indicated by an eventual drop in the platelet count to 50,000/mm^3^ blood or below. Our study shows a proof-of-concept that simple decision algorithms can predict dengue diagnosis and the likelihood of developing severe disease, a finding that could prove useful in the management of dengue patients and to public health efforts in preventing virus transmission.

## Introduction

Dengue fever/dengue haemorrhagic fever (DF/DHF) is a re-emerging disease throughout the tropical world. The disease is caused by four closely related dengue viruses, which are transmitted by the *Aedes* mosquitoes, principally *Aedes aegypti* [1]. DHF and dengue shock syndrome (DSS) represent the severe end of the disease spectrum, which if not properly managed, would result in significant mortality. The pathophysiology of severe DHF and DSS is characterized by plasma leakage as a result of alteration in microvascular permeability [2]. There is as yet no vaccine or specific antiviral therapy for DF/DHF and management of cases remains largely supportive [3].

Dengue illness is often confused with other viral febrile states, confounding both clinical management [4]–[6] and disease surveillance for viral transmission prevention [7]. This difficulty is especially striking during the early phase of illness, where non-specific clinical symptoms and signs accompany the febrile illness [4]. More definitive symptoms, such as retro-orbital pain, and clinical signs, such as petechiae, do not appear until the later stages of illness, if at all. Definitive early dengue diagnosis thus requires laboratory tests and those suitable for use at this stage of illness are either costly, such as RT-PCR for dengue; not sufficiently rapid, such as virus isolation; or undergoing field trials, such as ELISA for NS1 protein of dengue virus [8],[9]. Furthermore, many dengue endemic places lack the necessary laboratory infrastructure or support [7] and thus a scheme for reliable clinical diagnosis, using data that can be obtained routinely, would be useful for early recognition of dengue fever, not only for case management but also for dengue surveillance. The current World Health Organization (WHO) scheme for classifying dengue infection (Table S1) makes use of symptoms and signs that are often not present in the first few days of illness, and thus not a guide for early diagnosis, but are instead designed for monitoring disease progression for clinical management of the severe DHF/DSS. Other attempts at identifying clinical features for the diagnosis of dengue disease have made use of univariate or multivariate analysis of clinical symptoms and signs, haematological or biochemical parameters [10],[11]. Although such studies provide a list of symptoms and signs that could be associated with dengue disease, how these should be applied for clinical diagnosis is not apparent. Evidence-based triage strategies that identify individuals likely to have dengue infection in the early stages of illness are needed to direct patient stratification in clinical investigations, management and healthcare resource planning.

To address this goal, we show here that a decision tree approach can be useful to develop an intuitive diagnostic algorithm, using clinical and haematological parameters, that is able to distinguish dengue from non-dengue disease in the first 72 hours of illness. We also demonstrate a proof-of-concept that such an approach can be useful for early dengue disease prognostication.

## Materials and Methods

### Patients and clinical methods

#### Ethical considerations

The study protocol was approved by each organization's institutional review board. Patient enrolment, clinical and epidemiological data collection within the National Healthcare Group, Singapore was approved by the NHG IRB (DSRB B/05/013). Patient enrolment, clinical and epidemiological data collection in Dong Thap Hospital was approved by the hospital scientific and ethical committee as well as the Oxfordshire Tropical Research Ethical Committee, UK. Enrolment of study participants was conditional on appropriate informed consent administered by a study research nurse. All biological materials collected were anonymized after completion of demographic and clinical data collection.

#### Screening and recruitment

The protocol for patient recruitment in Singapore (the early dengue infection and outcome (EDEN) study) was described previously [12]. Adult patients (age >18 years) presenting at selected primary care polyclinics within 72 hours of onset of acute febrile illness and without rhinitis or clinically obvious alternative diagnoses for fever were eligible for study inclusion. Upon consent, anonymized demographic, clinical and epidemiological information were collected on a standardized data entry form on 3 occasions: 1–3 days post-onset of fever (1^st^ visit), 4–7 days post-onset of fever (2^nd^ visit) and 3–4 weeks post-onset of fever (3^rd^ visit). Venous blood was also collected for haematological, virological and serological analyses at every visit. Remaining serum and blood were anonymized and stored at −80°C until use. The list of parameters monitored in this study is shown in the supplementary Table S2.

Children or adults in whom there was a clinical suspicion of dengue were recruited within 72 hours of illness onset in Dong Thap Hospital, Vietnam. Blood samples were collected for diagnostic investigations at study enrolment and again at hospital discharge. Clinical data were collected daily on standard case record forms.

### Laboratory Methods

#### Haematology

A full blood count was performed on anticoagulated whole blood collected at all time points. A bench-top, FDA-approved haematocytometer was used for this application (iPoch-100, Sysmex, Japan). Calibration by internal and external QC controls was also performed on a regular basis.

#### Serology and antigen detection

IgM and IgG antibodies against dengue virus were detected using commercially available ELISAs (PanBio, Brisbane, Australia) according to manufacturer's instructions.

#### Reverse-transcription polymerase chain reaction (RT-PCR)

RNAs were extracted from the first serum portion or virus culture supernatant using QIAamp Viral RNA mini kit (Qiagen, Hilden, Germany) according to the manufacturer's protocol. RT-PCR to detect dengue viral RNA was carried out using a set of generic pan-dengue primers that targeted the 3′ non-coding region of dengue viruses as previously described [13]. Results were analysed with LightCycler software version 3.5 (Roche Diagnostics, Mannheim, Germany). Reactions with high crossover threshold (Ct) value or ambiguous melting curve results were analysed by electrophoresis on a 2% agarose gel, to confirm presence of product of the correct size. RNA extracted from previously obtained clinical isolates, namely dengue-1 (S144), dengue-2 (ST), dengue-3 (SGH) and dengue-4 (S006), propagated in C6/36 cell cultures were included as external control in every RT-PCR run.

### Decision tree analyses for disease modelling

#### Classifier modelling

The C4.5 decision tree classifier [14] software Inforsense (InforSense Ltd., London, UK) was used. A standard pruning confidence of 25% was used to remove branches where the algorithm was 25% or more confident so as to avoid having specific branches that would not be representative for generalisation. This prevents over-fitting of the data.

The parameter ‘minimal cases’ represents a stopping criterion for further partition of the data at specific decision nodes. Tree growing at a specific decision node was stopped when at least one class had equal or less cases than the ‘minimal cases’. This prevents the tree from sub-dividing into overly specific nodes which have little supporting data. Choosing an appropriate value for ‘missing cases’ was done using k-fold cross validation (see below). Briefly, various decision trees with different ‘minimal cases’ were calculated and the value resulting in the tree with the best performance was chosen.

The calculated algorithms were validated using the k-fold cross-validation approach. This is considered to be a powerful methodology to overcome data over-fitting [15]. Briefly, the original sample was divided into k sub-samples. Each sub-sample was put aside as evaluation data for testing a model, and the remaining k-1 sub-samples were used for training the model. The cross-validation process was repeated k times (folds) and each of the k sub-samples was used once as the validation data. The k results obtained from the k-folds could then be averaged to produce a single estimation of model performance [15]. The fold value was set to k = 10.

To analyse the sensitivity and specificity of the decision algorithm, an averaged receiver-operating characteristic (ROC) curve was constructed. The area under the curve (AUC) serves as an indicator of the overall performance of the algorithm. The algorithms with the highest sensitivity along with a high AUC were selected.

### Statistical analysis

All results have been summarized in terms of means and standard deviation for continuous variables using independent sample T-test. *Shapiro-Wilk* normality test was used to check for non-normally distributed parameters whereby a *p* value <0.05 indicated that the parameter was unlikely to originate from a normal distribution. Non-normally distributed parameters were log-transformed and rechecked for normality. If the log-transformation still resulted in non-normal distribution, non-parametric *Kruskal-Willis* test was used for continuous variables whereas *Student's* t test was exploited for normally distributed continuous variables. For dichotomous variables, *Chi-square* test was used in case of expected frequencies that were higher than 5, whereas *Fisher's* exact test was performed when the expected table values were smaller than 5. Cases with missing values were excluded from the analysis and thus, the number of cases used for calculations varied between different covariates. All calculations were performed using Systat for Windows (SYSTAT Software Inc. San Jose, CA). A two-tailed *p* value <0.05 was considered as statistically significant.

## Results

We constructed a decision tree for dengue diagnosis with 1,200 patients with acute febrile illness. Of these, 1,012 were recruited from the EDEN study and 188 from Vietnam. The EDEN cohort consisted of 173 DF, 3 DHF and 836 non-dengue cases while the Vietnam cohort consisted of 168 DHF and 20 DSS cases, resulting in a total of 364 dengue and 836 non-dengue cases used for our diagnostic tree construction.

**Figure 1:**
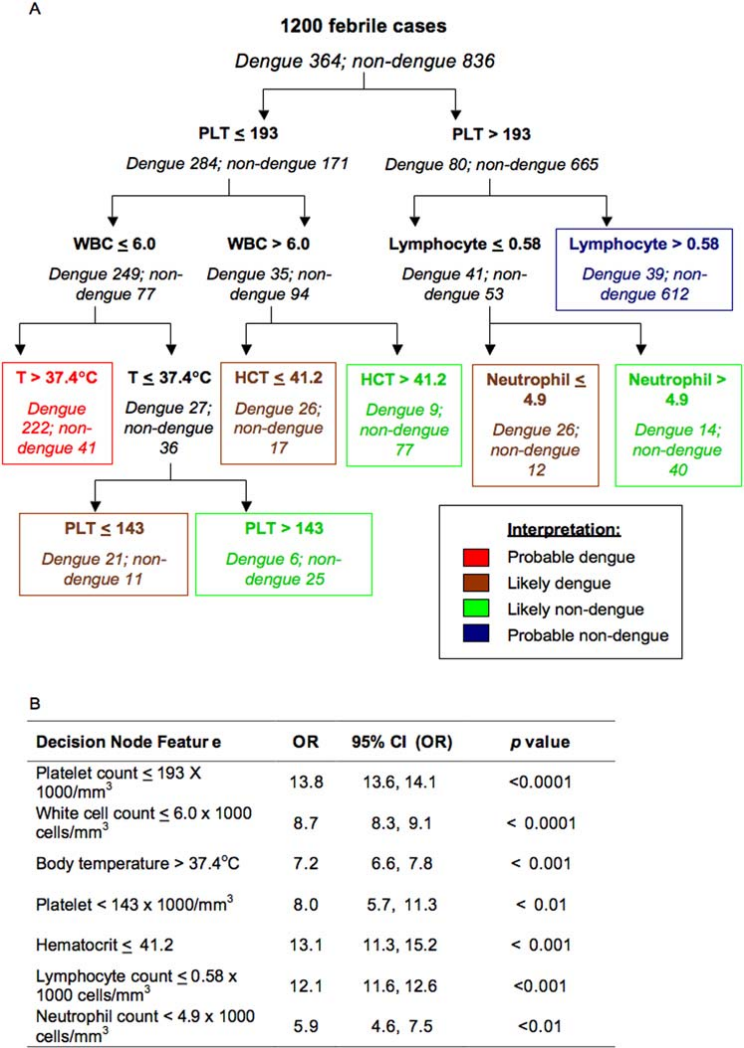
Decision algorithm for dengue diagnosis. A. Decision algorithm for predicting dengue diagnosis calculated on 1200 patients with data obtained in the first 72 hours of illness. PLT = *platelet count*; WBC = *white blood cell count*; T = *body temperature*; HCT = *hematocrit*; Lymphocyte = *absolute number of lymphocytes*; Neutrophil = *absolute number of neutrophils*. The prediction of the algorithm is shown in colours: Red indicates probable dengue; brown indicate likely dengue; green indicates likely non-dengue and blue indicates probably non-dengue. B. Statistical (chi-square) analysis of splitting criteria performed on each subgroup at the decision nodes. OR = *odds ratio*; CI = 95% *confidence interval.*

**Figure 2:**
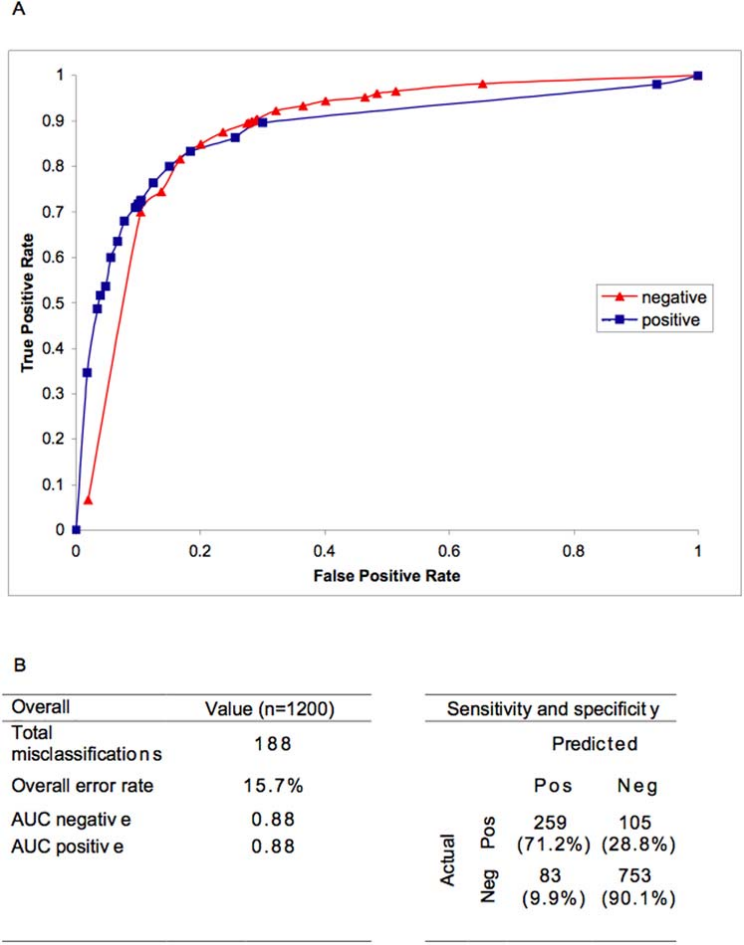
Performance of the decision algorithm for dengue diagnosis. A. Receiver operating characteristics (ROC) curve for the diagnostic algorithm in predicting dengue positive and dengue negative cases. B. Summary of K-fold (k = 10) cross-validation analysis for the dengue diagnostic algorithm with 2×2 analysis for the algorithm's sensitivity and specificity in dengue diagnosis.

The resulting diagnostic algorithm is shown in Figure 1. The first splitting parameter is a platelet count of 196,000/mm^3^ blood or less followed by the total white cell or lymphocyte counts, body temperature, haematocrit or neutrophil count and another platelet count at presentation. The predicted diagnosis is shown in colours, with red indicating probable dengue, brown indicating likely dengue, green indicating likely non-dengue and blue indicating probable non-dengue (Figure 1A). Each of the nodes showed statistical significance in the proportion of dengue and non-dengue cases, with the odds ratio calculated as shown in Figure 1B. The performance of this algorithm is shown in Figure 2. The overall error rate estimated after k-fold cross validation was 15.7%, with a sensitivity and specificity of 71.2% and 90.1%, respectively (Figure 2B).

### Prediction of disease severity

We also examined if the use of a decision tree would be useful for prognostication.

For the EDEN cohort, we used a platelet count of less than 50,000/mm^3^ on days 5 to 7 of illness as a marker of severe disease. This level of thrombocytopenia has been shown to be associated with the development of complications such as bleeding and shock in adults [16]–[18].

Fifteen cases were excluded from this analysis as they were either admitted to private hospitals where access to the clinical information was not available to us, or were foreigners who returned to their country of origin to seek medical treatment. Thus, 161 Singaporean dengue cases were analysed and the pruning confidence was set to 25% with minimal cases defined as 16.

**Figure 3:**
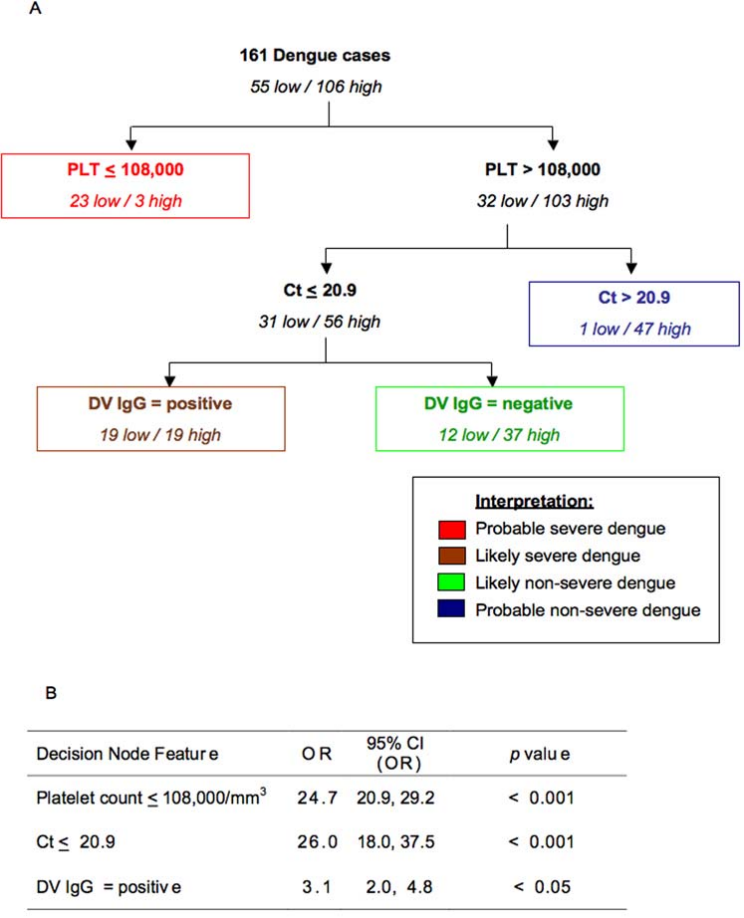
Decision algorithm for predicting severe dengue disease. A. Decision algorithm for severity prediction calculated on 169 patients with clinical data obtained at the first visit. PLT = *platelet count*; Ct  = *viral load whereby a high Ct-value indicates a low viral load;* DV IgG = *indicator for primary/secondary infection whereby a positive result indicates a secondary infection.* Low = *platelet nadir of 50,000/mm^3^ or less*; high = *platelet nadir greater than 50,000/mm^3^*. The prediction of the algorithm is shown in colours: Red indicates probably severe dengue; brown indicates likely severe dengue; green indicates likely non-severe dengue and blue indicates probable non-severe dengue B. Statistical (chi square) analysis of splitting criteria performed on each subgroup at the decision nodes. OR = *odds ratio*; CI = 95% *confidence interval.*. PLT = *platelet count*; Ct = *crossover threshold value of real-time RT-PCR and indicative of level of viremia;* DV IgG = *indicator for primary/secondary infection whereby a positive result indicates a secondary infection*; OR = *odds ratio*; CI = *confidence interval*.

**Figure 4:**
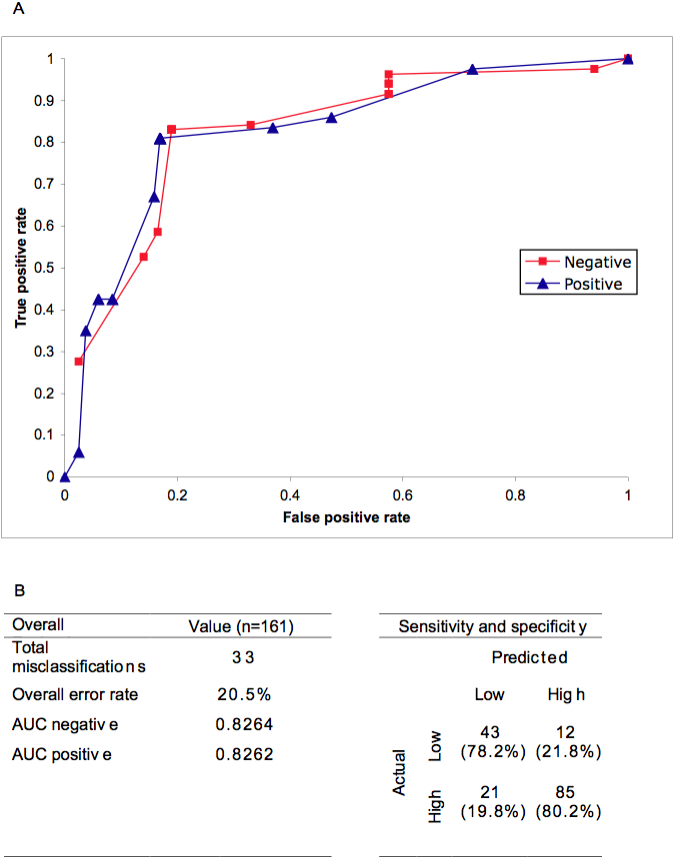
Performance of the decision algorithm for predicting severe dengue disease. A. Receiver operating characteristics (ROC) curve for the algorithm in predicting the development of severe disease among dengue cases. B. Summary of K-fold (k = 10) cross-validation for severity prediction algorithm with 2×2 analysis for the algorithm's sensitivity and specificity in predicting severe dengue disease.

The best performing decision algorithm made used of platelet count, the crossover value (Ct) of the real-time RT-PCR for dengue viral RNA (a marker for viremia levels) and the presence of anti-dengue IgG antibodies, as the first, second and third splitting parameters, respectively (Figure 3). All 3 parameters were obtained from the first visit. All three DHF cases were correctly classified using this algorithm, one into the group with a platelet count of 108,000 mm^3^ or less, the other two into the group with pre-existing anti-dengue IgG antibodies. The predicted outcome of disease is shown in colours, with red indicating probable severe dengue, brown indicating likely severe dengue, green indicating likely non-severe dengue and blue indicating probable non-severe dengue (Figure 3A). The statistical significance of each node of the algorithm and their odds ratio with severe dengue are shown in Figure 3B. The performance of this algorithm is shown in Figure 4. The overall error rate using k-fold crossover validation analysis was 20.5%, with a sensitivity of 78.2% and specificity of 80.2% (Figure 4B).

The use of data obtained from the 89 hospitalised cases alone resulted in a very similar decision algorithm, although the AUCs were substantially lower than the above analysis due largely to the smaller dataset. Taken together, these indicate that the prediction algorithm as defined in Figure 3A is stable.

We next examined the clinical outcomes of the patients grouped according to the decision algorithm in Figure 3A. The results are summarised in Table 1. Each of the four groups of patients showed different rates of hospitalisation, duration of hospitalisation and the proportion of clinically severe cases. The latter was defined as patients who met the criteria for DHF; had a systolic blood pressure less than 90 mmHg; a serum transaminase of greater than 1000 which suggests severe liver involvement and who received blood transfusion. The results indicate that statistically significant differences were observed between the groupings as indicated in Table 1.

**Table 1:** Number of cases hospitalised; mean number of days hospitalised and the number of clinically severe dengue cases from the EDEN cohort, grouped according to the dengue case prognosis algorithm shown in Figure 3A.

Prognostic tree grouping	No. of cases hospitalised (%)	Mean number of days hospitalised	SD (days)	No. of clinically severe cases (%)†
Probable severe dengue (n = 26)	25 (96.2)	5.2	1.4	10 (38.5)
Likely severe dengue (n = 38)	26 (63.4)	4.9	1.6	9 (23.7)
Likely non-severe dengue (n = 49)	27# (55.1)	3.9*	1.2	4 (8.2)#
Probable non-severe dengue (n = 48)	10** (20.8)	3.5**	1.2	0#

**†:** indicates cases with DHF/SBP<90mmHg/serum transaminase>1000/transfusion.

*: p<0.05 and ** p<0.001 when compared to either probable severe dengue or likely severe dengue. # indicates p<0.05 when compared to the probable severe dengue only.

## Discussion

The lack of evidence-based diagnostic algorithm for early dengue diagnosis as well as prognostic triage strategies limits effective patient management, use of healthcare resources and disease surveillance efforts. For instance, over 80% of the total dengue cases in Singapore are admitted for hospitalised care, mostly to monitor for signs of clinical deterioration. Prognostication in the early stages of dengue illness could significantly influence clinical management and the use of healthcare resources, particularly during dengue outbreaks, such as occurred in Singapore in 2005 where up to 8% of all acute hospitals beds available were occupied by dengue cases [19].

To identify the decision algorithms, we have used a C4.5 decision tree classifier, which has several advantages over other statistical tools [14]. Briefly, decision algorithms are in principle simple to understand and are able to handle both nominal and categorical data. Importantly, they are also able to handle missing values, which are commonly encountered in clinical studies. In contrast, logistic regression and discriminant analyses require much more data preparation and appropriate handling of missing values for reliable calculations [15]. Decision algorithms are also easy to interpret, use and validate using common statistical techniques. Importantly, it provides a means to identify parameters that would be significantly associated with disease when analysed in sub-groups but not in the total study population. To our understanding, this is the first time decision tree modelling has been used to identify prognostic markers for dengue disease.

While dengue is predominantly a paediatric disease, dengue in adults has become an increasingly recognised problem, both in dengue-endemic regions [6],[20] as well as in adult travellers returning from the tropics [5]. The case recruitment in Singapore has thus focused on adult cases. Since the course of disease in all but three of the Singaporean adult cases were consistent with DF instead of DHF, we have included 188 DHF/DSS paediatric and adult cases from Vietnam in order to ensure that the diagnostic algorithm developed here is robust across a spectrum of dengue presentations.

The decision algorithm for the diagnosis of dengue within the first three days of illness made use of a combination of platelet count, total white cell count, body temperature, absolute lymphocyte and neutrophil counts, in sequential order (Figure 1A). Each node of the decision tree has statistically significant odds ratio ranging from 5.9 to 13.8 (Figure 1B).

Although the tree has an optimal combined sensitivity and specificity of 71.2% and 90.3%, respectively, its usage can be adjusted according to the objective in which it is used for. In an outbreak where the aim is to identify all dengue cases for laboratory investigation and clinical follow-up, the tree could be used to exclude dengue cases whereupon all cases except those predicted as probable non-dengue (shown in blue in Figure 1A) are tested for dengue virus. When applied hypothetically to an outbreak similar to that observed in Singapore in 2005 where 29% of the acute febrile cases recruited into our study was dengue, the positive and negative predictive values of the tree are 57.7% and 94.4%, respectively. Conversely, when the dengue prevalence is low as was encountered in our EDEN study between 2006 and August 2007 where only 43 out of 555 (7.7%) cases presenting with acute febrile illness were dengue, increasing the specificity of clinical diagnosis by selecting patients with probable dengue (shown in red in Figure 1A) would result in a positive and negative predictive values of 51.1% and 97.7%, respectively. Such a level of positive predictive value would be useful to guide the selection of patients for virological surveillance, a critical part of any dengue prevention program [7], [21]–[23].

Upon diagnosis, current dengue management strategies require daily observation for signs of clinical deterioration, particularly for clinical or laboratory evidence of hemorrhage or plasma leakage. In situations of high prevalence of dengue illness, such an approach can quickly overwhelm limited healthcare resources. It would be advantageous to be able to stratify dengue cases for clinical follow-up and management based on the likely outcome of disease. We thus searched for an algorithm that could be used for prognostication. Since the incidence of DHF is low in Singapore [20] and hospitalisation of the dengue cases is subject to variation arising from physician-to-physician differences in decision-making, we have used platelet count nadir of 50,000/mm^3 ^or less at 5 to 7 days after onset of illness as an objective end-point for our analysis. This level of thrombocytopenia has been found to be associated with increased risks of haemorrhage and shock in adults with DF [16]–[18]. We were unable to include the DHF and DSS cases recruited in Vietnam for the tree construction as daily laboratory parameters comparable to those collected for the Singapore cohort were not available.

The decision algorithm for prognostication (Figure 3A) uses the platelet count as the first splitting criteria, followed by the dengue virus genome copy number estimated by real-time RT-PCR as the second splitting criteria for those with platelet count greater than 108,000/mm^3^ blood. The second splitting criterion is a marker of viral load. Although we have used the Ct value of our real-time RT-PCR in this analysis, it is likely that other parameters that provide estimates for the viral load could be substituted for the viral genome copy numbers. The development of NS1 antigen ELISA that is currently being evaluated in several places could be one such alternative. The third splitting criterion uses the presence of anti-dengue IgG antibody, indicating secondary infection.

Although thrombocytopaenia [16]–[18], high viremia [24] and presence of pre-existing anti-dengue antibodies [24]–[26] have previously been reported to be associated with severe disease, how these factors should be used clinically for prognostication has never been described. Furthermore, using these parameters singly also presents difficulties since these parameters are dynamic and the window period in which these parameters offer peak predictive values is extremely short [9]. The use of a decision tree approach could thus provide clinicians with an algorithm to guide the evaluation of a panel of critical laboratory parameters and the sequential order these should be considered within the first 72 hours of illness.

Each of the four groups of patients under the decision algorithm for prognosis (Figure 3A) also showed significant differences in clinical outcome (Table 1). While only three cases met all the criteria for DHF according to the WHO dengue classification, the clinical records of another 20 cases showed that they either had a period of hypotension (systolic blood pressure of less than 90mmHg) or severe liver inflammation (liver transaminases>1000), both without documented pleural effusion, ascites or rise in serial hematocrit, or received platelet/blood transfusion. These clinical parameters have been previously observed in severe dengue [15],[16] and we have taken these cases collectively as clinically severe outcomes. Of these 23 cases, 19 (82.6%) were predicted by our tree as either probable severe dengue or likely severe dengue with data obtained in the first three days of illness. Conversely, 91.8% and 100% of the patients in the groups predicted by our tree as either likely non-severe dengue or probable non-severe dengue, respectively, did not show severe clinical outcomes (Table 1).

The use of such a prognostic algorithm could prove useful in segregating patients according to likely clinical outcomes to guide clinical management and follow-up visits. Although our EDEN cohort in Singapore has focused on dengue in the adult population, our findings demonstrate a proof-of-concept that the use of simple haematological and virological parameters is predictive of disease outcome, and can be built upon to develop prognosis-based protocols for dengue case management that begins at the primary healthcare setting.

Our study represents the first to demonstrate that decision algorithms for dengue diagnosis and prognosis can be developed for clinical use. While a large multi-centre prospective study will be needed for these algorithms to be applied globally, our analysis indicates that a decision tree approach can differentiate dengue from non-dengue febrile illness and predict outcome of disease.

## Supporting Information

Table S1 Criteria for the classification of DF/DHF and the recommended approach to diagnosis, according to the WHO Guidelines.
(0.03 MB DOC)

Table S2 Parameters and the respective units of measure used in the EDEN study to monitor the recruited cases in all three visits.
(0.06 MB DOC)
