# Supplementary material for: Decision Tree Algorithms Predict the Diagnosis and Outcome of Dengue Fever in the Early Phase of Illness
Source: PLoS Negl Trop Dis. 2008 Mar 12;2(3):e196. doi: 10.1371/journal.pntd.0000196 (PMC2263124; doi:10.1371/journal.pntd.0000196)
Supplement: Table S1 — Criteria for the classification of DF/DHF and the recommended approach to diagnosis, according to the WHO Guidelines. (0.03 MB DOC) [file pntd.0000196.s001.doc]

**Table S1.** Criteria for the classification of DF/DHF and the recommended approach

to diagnosis, according to the WHO Guidelines.

| **DF** | **DHF** | **Diagnostic approach** |
| --- | --- | --- |
| Acute febrile illness with 2 or more of the following manifestation:   - headache - retro-orbital pain - myalgia - arthralgia - rash - haemorrhagic manifestation - leukopaenia   with necessary laboratory confirmation. | The following must be present:   - Acute fever lasting 2-7 days - Haemorrhagic tendency - Thrombocytopenia (100,000 cells/mm^3^ or less) - Evidence of plasma leakage | Clinical indicators of DHF   - High fever of acute onset - Haemorrhagic manifestations - Hepatomegaly - Shock   Laboratory indicators of DHF   - Thrombocytopenia (100,000 cells/mm^3^ or less) - Haemoconcentration (haematocrit elevated at least 20% above average for age, sex and population) |
