# Supplementary material for: Decision Tree Algorithms Predict the Diagnosis and Outcome of Dengue Fever in the Early Phase of Illness
Source: PLoS Negl Trop Dis. 2008 Mar 12;2(3):e196. doi: 10.1371/journal.pntd.0000196 (PMC2263124; doi:10.1371/journal.pntd.0000196)
Supplement: Table S2 — Parameters and the respective units of measure used in the EDEN study to monitor the recruited cases in all three visits. (0.06 MB DOC) [file pntd.0000196.s002.doc]

**Table S2.** Parameters and the respective units of measure used in the EDEN study to

monitor the recruited cases in all three visits.

| **Parameter** | **Abbreviation used** | **Unit of measure** |
| --- | --- | --- |
| Body temperature | TEMP | ^o^C |
| Pulse rate |  | Beats per minute |
| Systolic blood pressure | Systolic BP | mmHg |
| Diastolic blood pressure | Diastolic BP | mmHg |
| White blood cell count | WBC | Cells/microlitre |
| Red blood cell count | RBC | Cells/microlitre |
| Haemoglobin concentration | HGB | g/dL |
| Haematocrit | HCT | % |
| Mean corpuscular volume | MCV | Femtolitre |
| Mean corpuscular haemoglobin | MCH | pg/cell |
| Mean corpuscular haemoglobin concentration | MCHC | g/dL |
| Platelet count | PLT | /mm^3^ |
| Percentage of lymphocyte in WBC | LYMPH% | % |
| Percentage of monocytes, eosinophils and basophils in WBC | MXD% | % |
| Percentage of neutrophils in WBC | NEUT% | % |
| Lymphocyte count | LYMPH No | Absolute number |
| Monocyte, eosinophil and basophil count | MXD No | Absolute number |
| Neutrophil count | NEUT No | Absolute number |
| Red cell distribution width coefficient of variation | RDW CV | % |
| RDW CV standard deviation | RDW SD | % |
| Platelet distribution width | PDW | Femtolitre |
| Mean platelet volume | MPV | Femtolitre |
| Platelet large cell ratio | PLCR | % |
| Dengue virus real-time RT-PCR | DV RT-PCR | Positive/negative |
| Crossover threshold of DV RT-PCR | Ct | Number of cycles |
| Anti-dengue IgG antibodies | DV IgG | Positive/negative |
|  |  |  |
| Symptoms and signs |  |  |
| Drowsiness |  | Reported/not reported |
| Headache |  | Reported/not reported |
| Muscle pain |  | Reported/not reported |
| Joint pain |  | Reported/not reported |
| Loss of appetite |  | Reported/not reported |
| Diarrhoea |  | Reported/not reported |
| Nausea |  | Reported/not reported |
| Vomiting |  | Reported/not reported |
| Red eyes |  | Reported/not reported |
| Rashes |  | Reported/not reported |
| Retroorbital pain |  | Reported/not reported |
| Taste alteration |  | Reported/not reported |
| Skin sensitivity |  | Reported/not reported |
| Bleeding |  | Reported/not reported |
| Palpable lymphadenopathy |  | Reported/not reported |
